# Supplementary material for: Altered chromatin landscape and enhancer engagement underlie transcriptional dysregulation in MED12 mutant uterine leiomyomas
Source: Nat Commun. 2020 Feb 24;11:1019. doi: 10.1038/s41467-020-14701-6 (PMC7040020; doi:10.1038/s41467-020-14701-6)
Supplement: Supplementary file 2 — Supplementary Information [file 41467_2020_14701_MOESM2_ESM.pdf]

Supplementary Information for:

Altered chromatin landscape and enhancer engagement underlie transcriptional dysregulation in MED12 mutant uterine leiomyomas

Moyo et al.

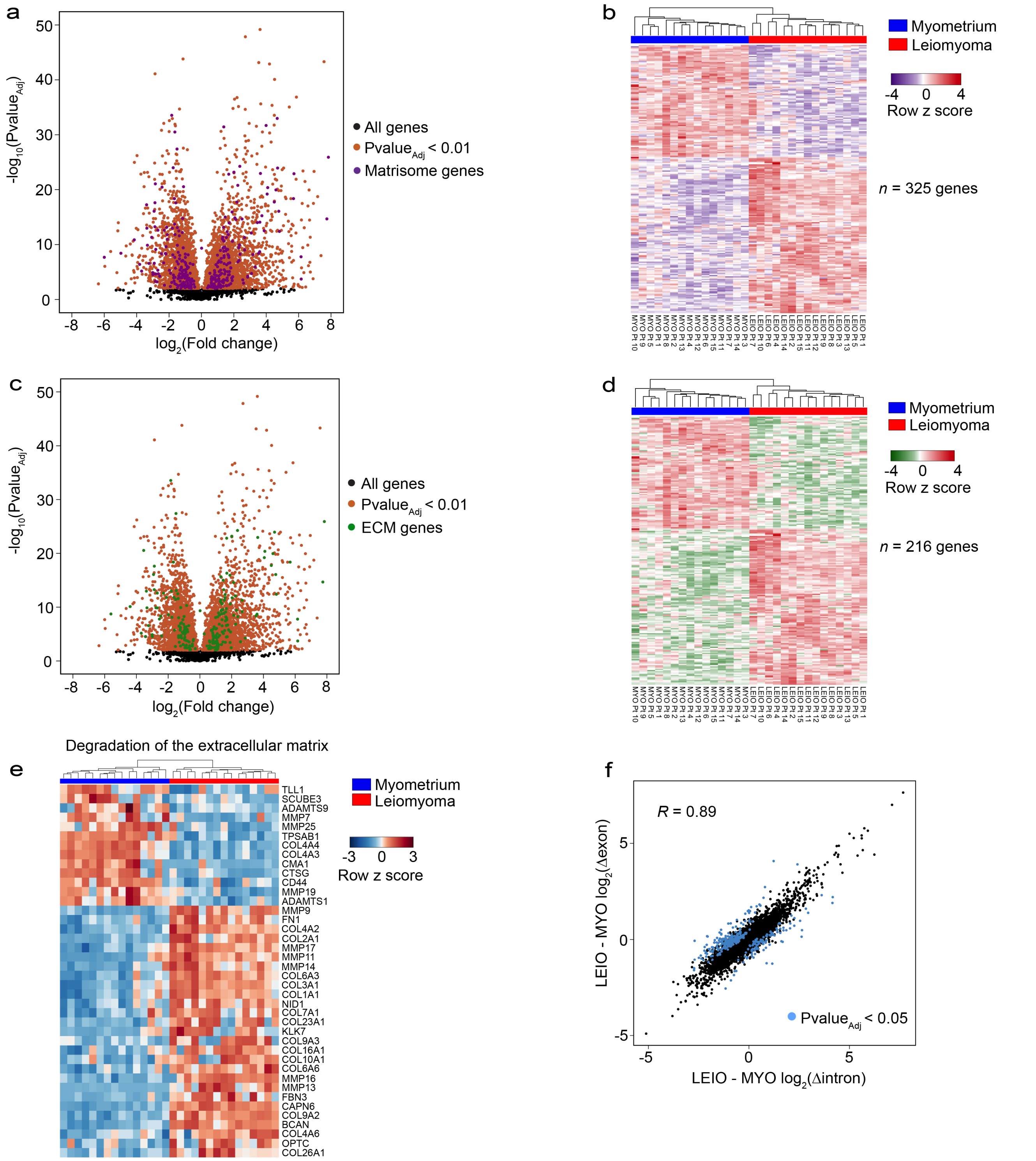

Supplementary Figure 1. Transcriptome profiling reveals transcriptional dysregulation of extracellular matrix associated genes. (a) Volcano plot of expression levels in patient tissue samples of all transcribed genes (black) in the human genome. Differentially expressed genes (DEGs) (sienna, n = 5,831) and DEGs associated with the core matrisome (GO:M5884) ontology term (purple, n = 325), which comprises extracellular matrix (ECM) and ECM-associated genes, are highlighted. P values are truncated at 1×10^{-50} for visualization purposes (FDR < 0.01, BH corrected Wald test). **(b)** Heat map of all differentially expressed core matrisome genes. Gene expression levels relative to the mean expression are shown as row z scores (n = 325 genes). **(c)** Volcano plot of DEGs (sienna, n = 5,831) and DEGs associated with the production and regulation of the ECM (green, n = 216), which is a subset of the matrisome ontology term, are highlighted. P values are truncated at 1×10^{-50} for visualization purposes (FDR < 0.01, BH corrected Wald test). **(d)** Heat map of all DEGs associated with the production and regulation of the ECM. Gene expression levels relative to the mean expression are shown as row z scores (n = 216 genes). **(e)** Heat map of a subset of differentially expressed ECM genes associated with the degradation of the ECM (R-HSA-1474228) ontology term. **(f)** Scatter plot of exon-intron split analysis (EISA) comparing changes in exonic reads vs. changes in intronic reads of all expressed genes between myometrium and leiomyoma tissues. R represents Pearson correlation coefficient.

IP	Total # of Peaks	# of differential Peaks
**H3K27Ac**	54,488	16,752
**RNAPII**	34,840	6,163
**JUN**	18,283	2,919
**FOS**	25,736	2,993
**CDK8**	44,402	4,696
**MED12**	52,033	6,857
**CDK8 submodule (CDK8-MED12 overlap)**	30,697	5,409

Supplementary Table 1. Total ChIP consensus peaks identified from all myometrium and leiomyoma biological replicates for H3K27Ac, RNAPII, JUN, FOS, CDK8 and MED12. Numbers of differential peaks for each IP are also noted. Total consensus peaks and differential peaks that are co-bound by CDK8 and MED12 (CDK8 submodule) are also listed.

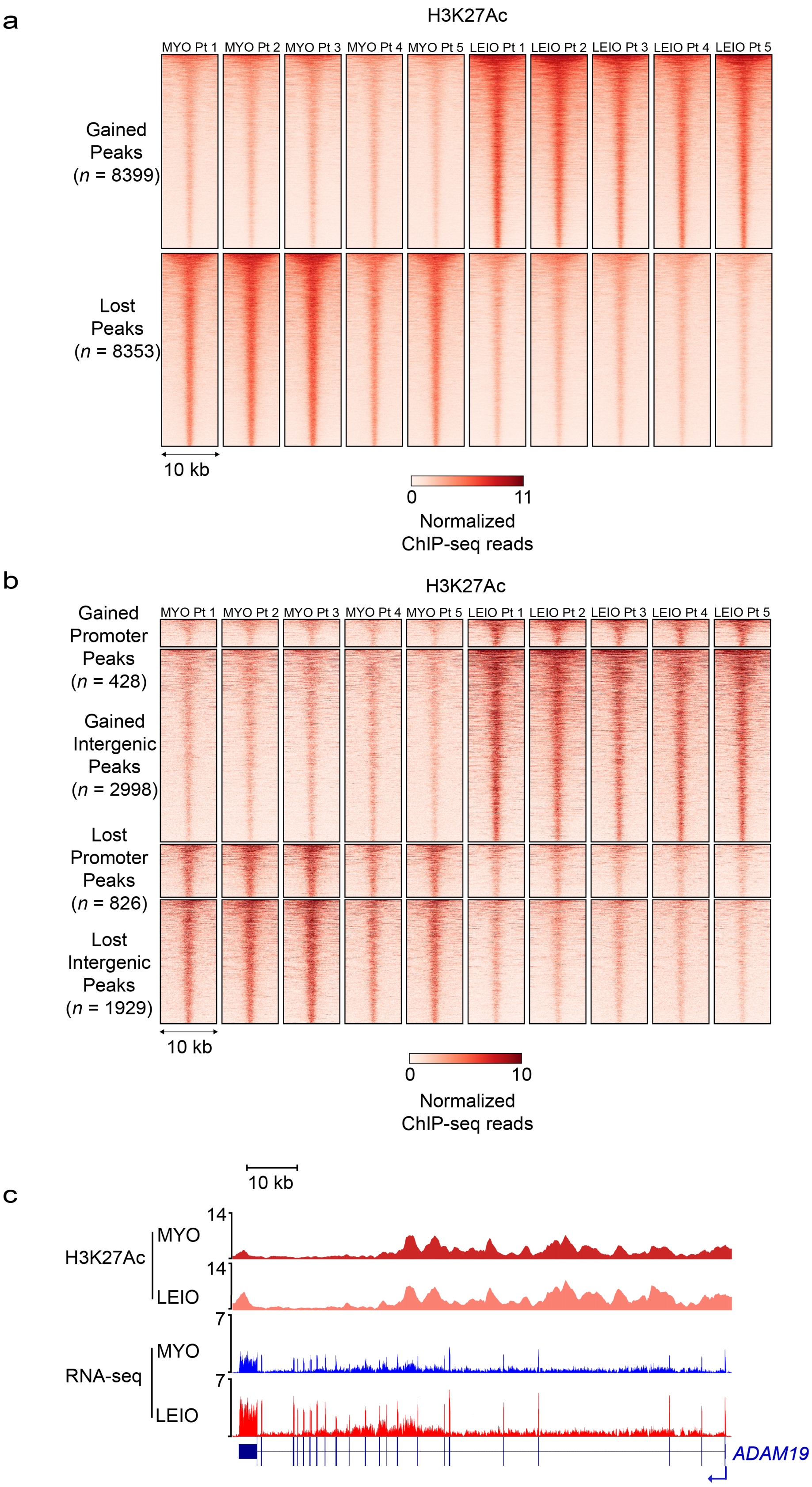

Supplementary Figure 2. Histone acetylation changes occur disproportionately at intergenic regions. **(a)** Heat map of normalized H3K27Ac ChIP-seq reads at differentially acetylated regions in myometrium vs. leiomyoma. Signal for each biological replicate is shown. **(b)** Heat map of normalized H3K27Ac ChIP-seq reads at gene promoters and intergenic loci with differential H3K27Ac signal. Signal for each biological replicate is shown. **(c)** Unchanged H3K27Ac signal in myometrium (red) and leiomyoma (salmon) at *ADAM19*. Normalized RNA reads for myometrium (blue) and leiomyoma (red) are also shown.

	Total # of contacts	# of altered contacts
**Enhancer-Promoter (E-P)**	163,712	8,078
**Promoter-Promoter (P-P)**	27,392	1,428

Supplementary Table 2. Promoter capture Hi-C contacts identified from all myometrium and leiomyoma biological replicates. Enhancer-promoter (E-P) and Promoter-promoter (P-P) contacts are listed, with altered contacts also divided into E-P and P-P contact groups.

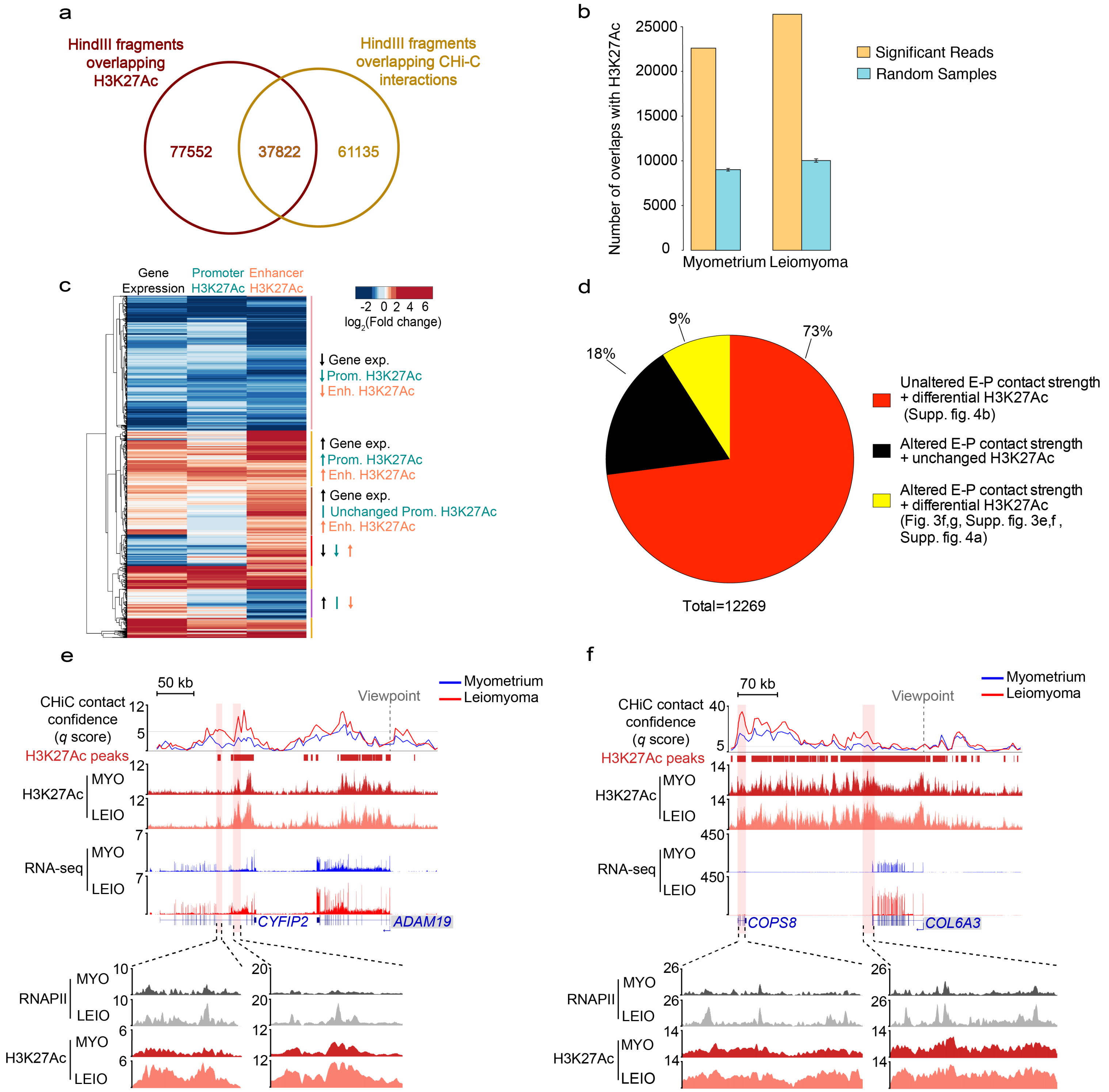

Supplementary Figure 3. Enhancer malfunction leads to gene expression changes in leiomyomas. (a) Venn diagram of promoter contacts overlapping all acetylated H3K27 regions in myometrium and leiomyoma tissue. HindIII digest fragments of the human genome are used to define unique, non-overlapping regions in the genome, to which H3K27Ac peaks and E-P contacts are then assigned. See CHi-C data processing and analysis in methods. **(b)** Bar graph of H3K27Ac peak enrichment in regions containing E-P contacts. Expected number of H3K27Ac peaks (Random Samples) that would overlap with E-P contacts by chance (blue bars, 95% confidence interval) and the observed H3K27Ac peaks that overlap with E-P contacts in myometrium and leiomyoma (orange bars) are shown. **(c)** Heat map of changes in gene expression, promoter H3K27Ac, and enhancer H3K27Ac of differentially expressed genes. Genes are hierarchically clustered by similarity in profiles of differential gene expression and H3K27 acetylation (n = 3,183) **(d)** Pie chart of modified cis-regulatory promoter-distal regions classified according to H3K27 acetylation status and alterations in promoter contact strength. **(e, f)** Genomic loci for *ADAM19* (e) and *COL6A3* (f) genes; both associated with altered promoter contacts. Altered confidence scores (CHiCAGO q scores) are shown for myometrium and leiomyoma. In addition H3K27Ac ChIP-seq and RNA-seq genomic tracks are shown, with H3K27Ac and RNAPII ChIP-seq signal in regions containing altered contacts highlighted (bottom zoomed in insert).

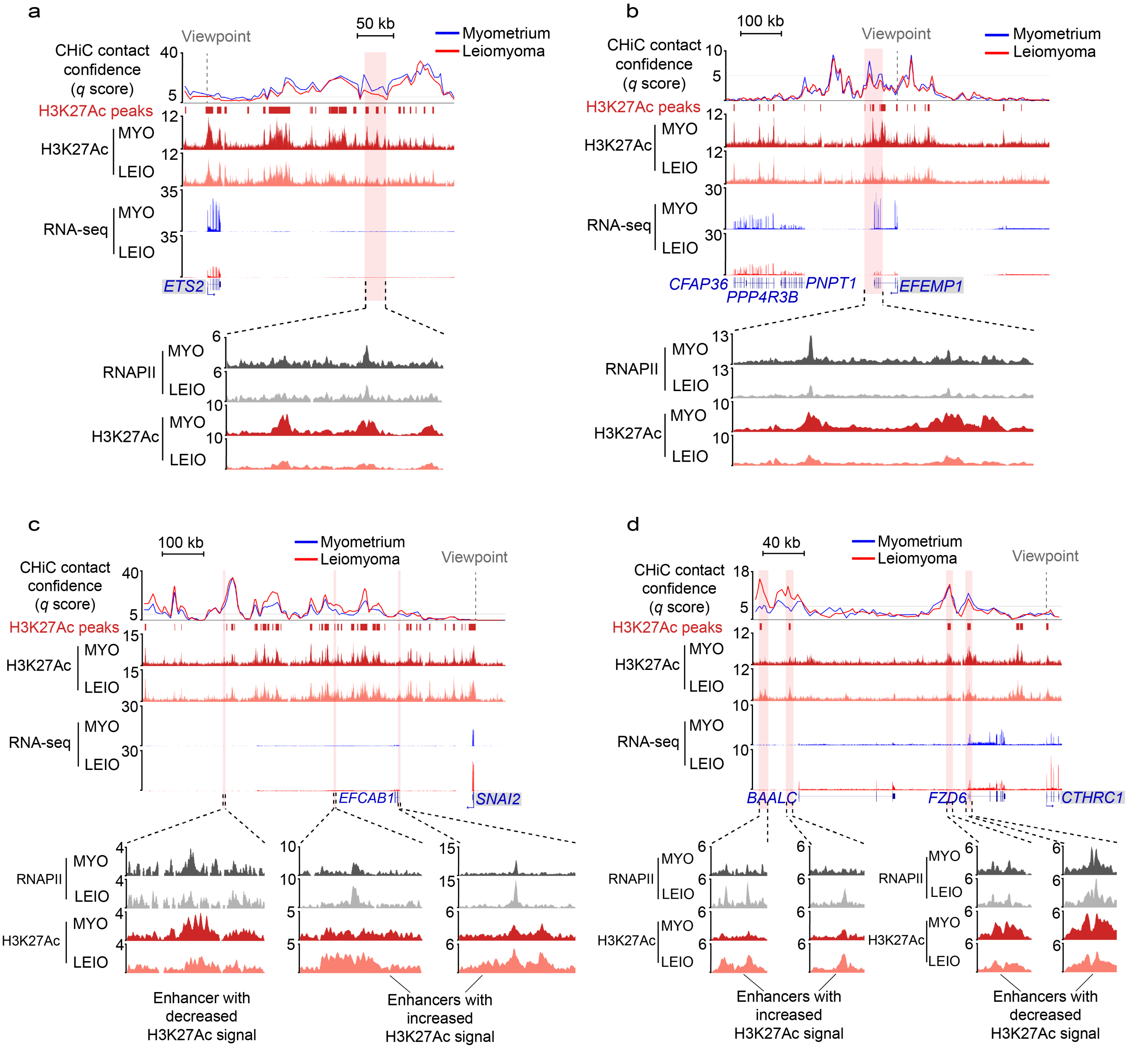

Supplementary Figure 4. Differential enhancer usage occurs between multiple enhancers associated with the same differentially expressed genes. (a, b) Genomic loci for *ETS2* (a) and *EFEMP1* (b) genes. Altered enhancer architecture involving changes to both H3K27Ac signal and promoter contact strength (a) or changes in H3K27Ac signal at enhancers with stable, unchanging promoter contact strength (b). **(c, d)** Genomic loci for *SNAI2* (c) and *CTHRC1* (d), both of which exhibit cases of differential enhancer usage. Differential enhancer usage generally involves changes in distal regions with stable enhancer-promoter contacts and differential H3K27Ac signal (c) but may involve enhancers with altered promoter strength as well as differential H3K27Ac signal (d).

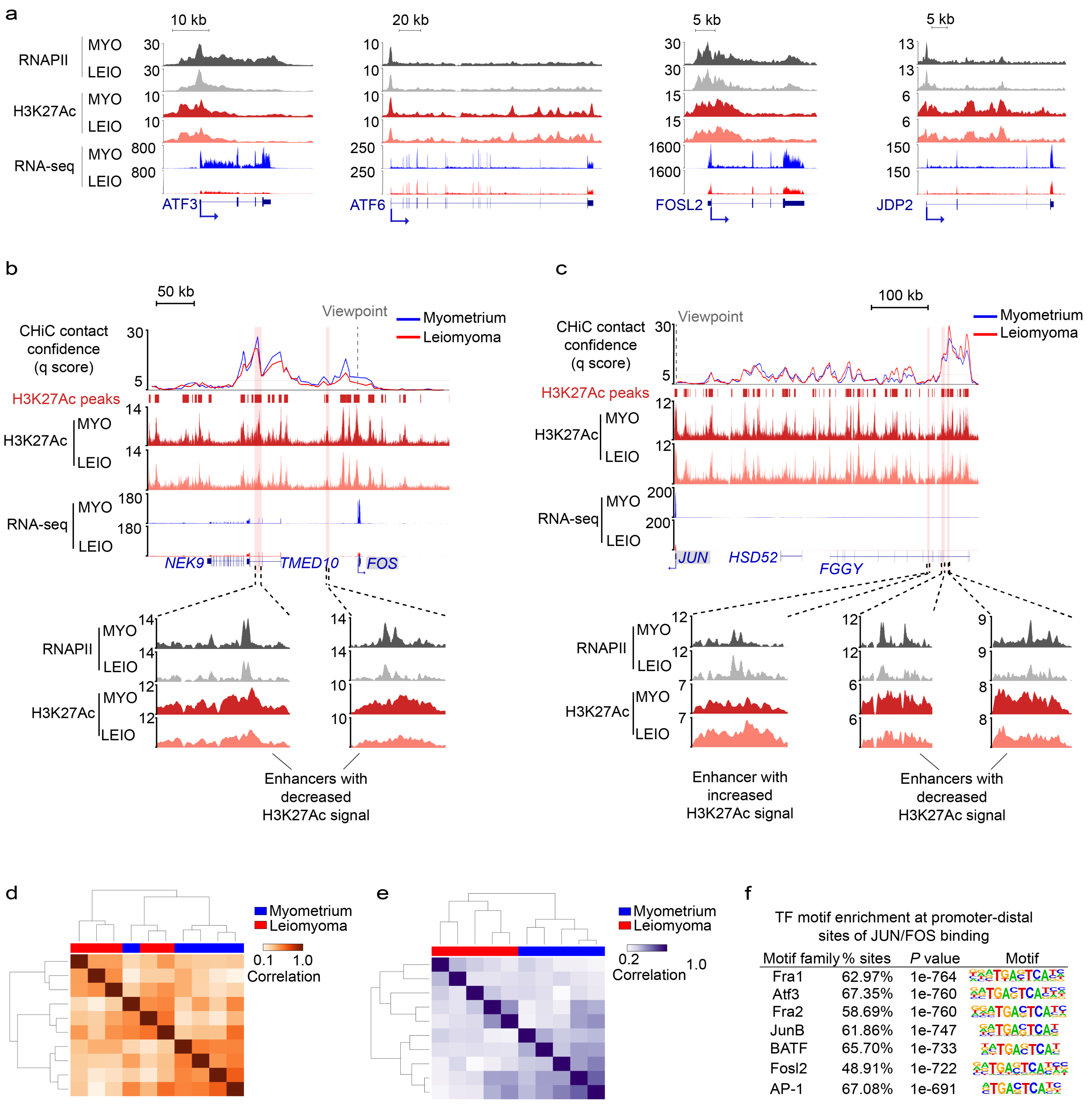

Supplementary Figure 5. AP-1 expression and chromatin occupancy is perturbed in leiomyomas. (a) H3K27Ac, RNAPII ChIP-seq and RNA-seq genomic tracks for AP-1 subunits *ATF3*, *ATF6*, *FOSL2*, *JDP2*. **(b, c)** Genomic loci for *FOS* (b) and *JUN* (c) showing RNA expression and enhancer-promoter contacts in myometrium and leiomyoma. H3K27Ac and RNAPII ChIP-seq at sites of increased or decreased H3K27Ac signal are highlighted. **(d, e)** Correlation (Pearson) heat map of FOS (d) and JUN (e) ChIP sample affinity scores obtained from myometrium (blue) and leiomyoma (red) ChIP-seq read counts. **(f)** Top identified transcription factor motifs (P value ranking, binomial) enriched in enhancer regions bound by FOS or JUN in myometrium vs. leiomyoma.

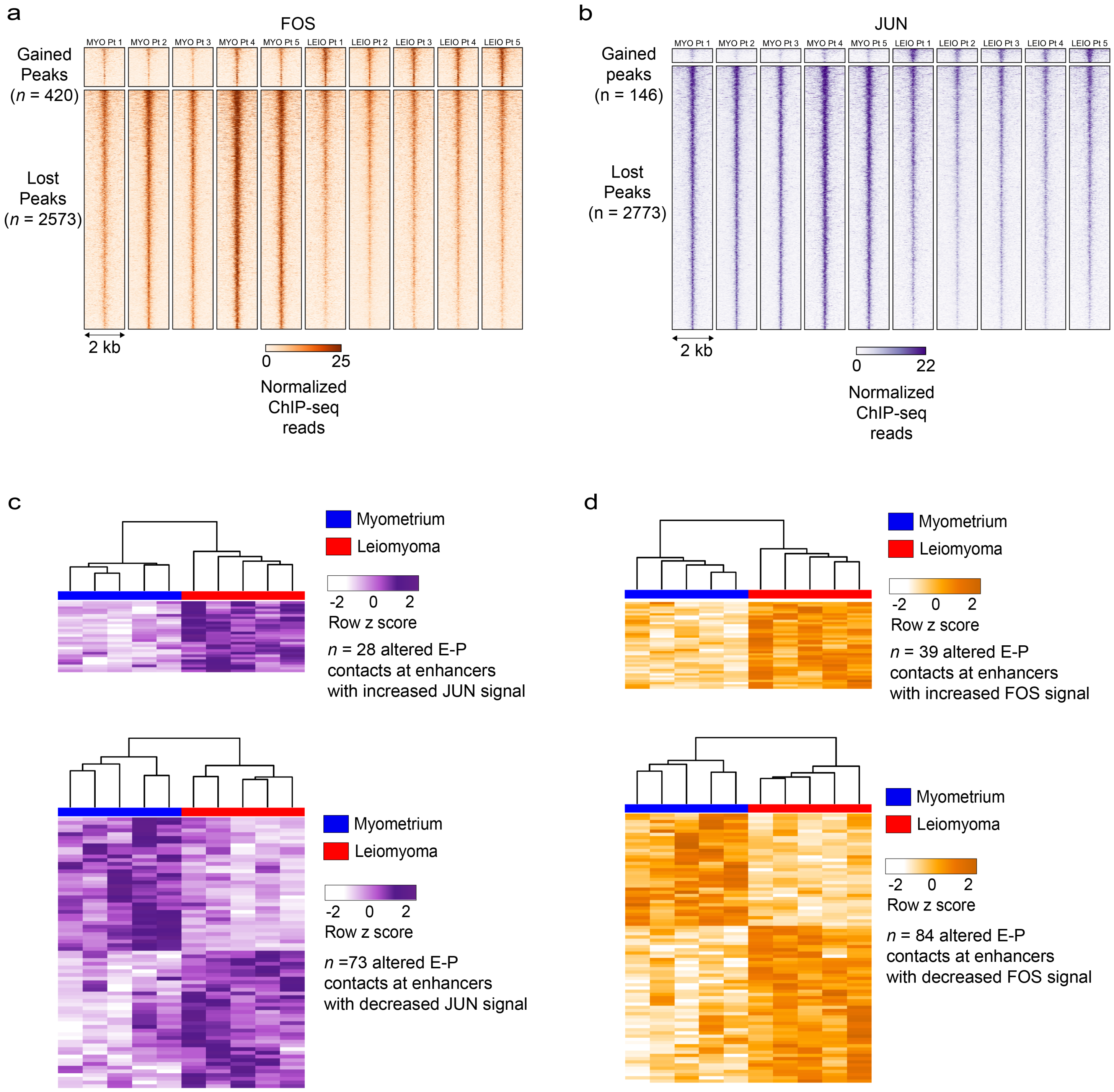

Supplementary Figure 6. AP-1 driven modifications in enhancer architecture result in differential gene expression. (a, b) Heat map of normalized FOS (a) and JUN (b) ChIP-seq reads at differentially bound *FOS* and *JUN* regions respectively in myometrium vs. leiomyoma. Signal for each biological replicate is shown. **(c)** Heat maps of altered enhancer-promoter contacts that overlap with enriched JUN (top panel, n = 28 contacts) and depleted JUN (bottom panel, n = 73 contacts) at enhancer regions in myometrium and leiomyoma tissues. **(d)** Heat maps of altered enhancer-promoter contacts that overlap with enriched FOS (top panel, n = 39 contacts) and depleted FOS (bottom panel, n = 84 contacts) at enhancer regions in myometrium and leiomyoma tissues.

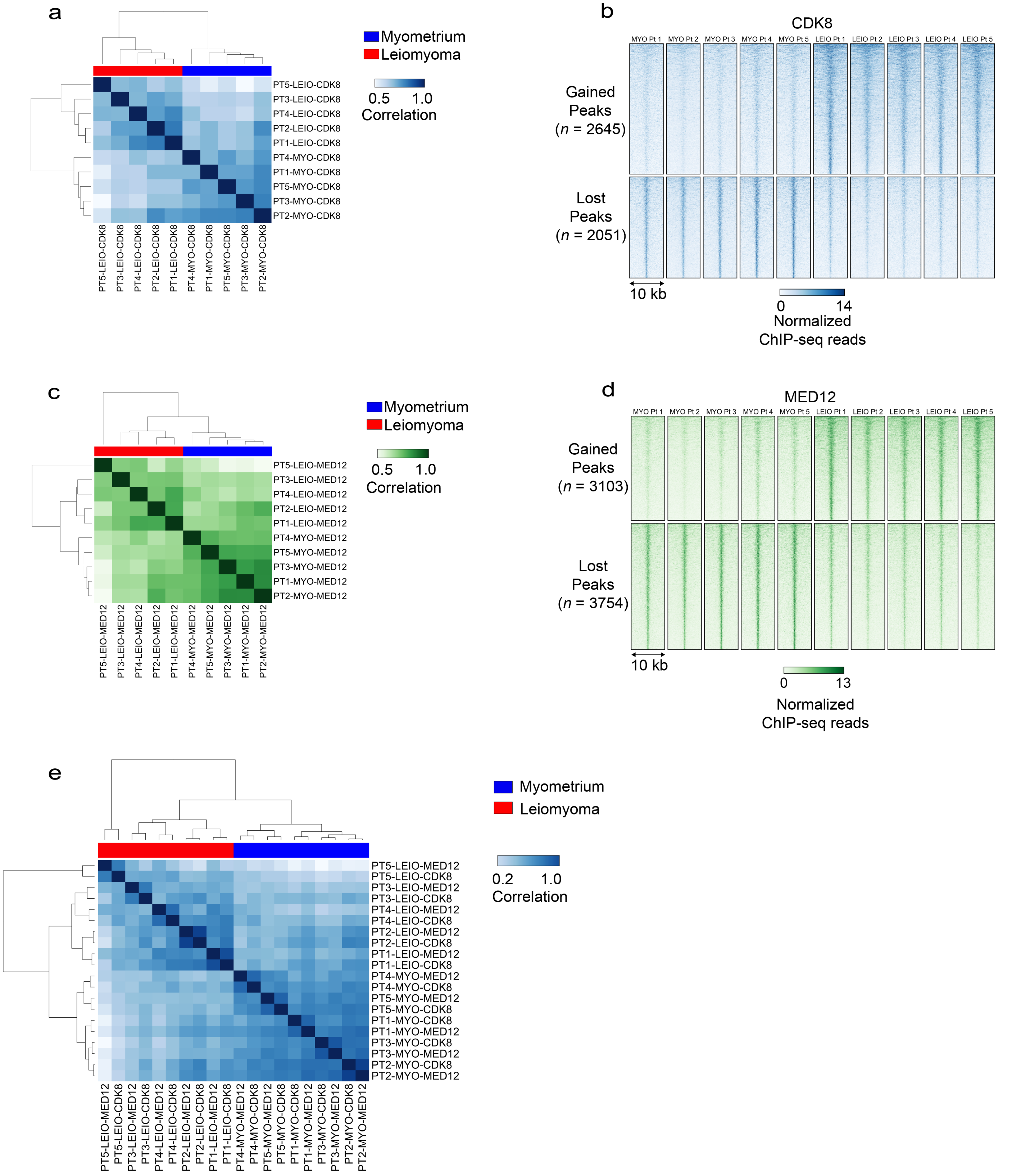

Supplementary Figure 7. CDK8 subcomplex chromatin occupancy correlates with changes in enhancer acetylation. (a, c) Correlation (Pearson) heat map of CDK8 (a) and MED12 (c) ChIP sample affinity scores obtained from myometrium (blue) and leiomyoma (red) ChIP-seq read counts. **(b, d)** Heat map of normalized CDK8 (b) and MED12 (d) ChIP-seq reads at differentially bound CDK8 and MED12 regions respectively in myometrium vs. leiomyoma. Signal for each biological replicate is shown. (e) Correlation (Pearson) heat map of CDK8 and MED12 ChIP sample affinity scores at sites co-bound by CDK8 and MED12 in myometrium (blue) and leiomyoma (red).

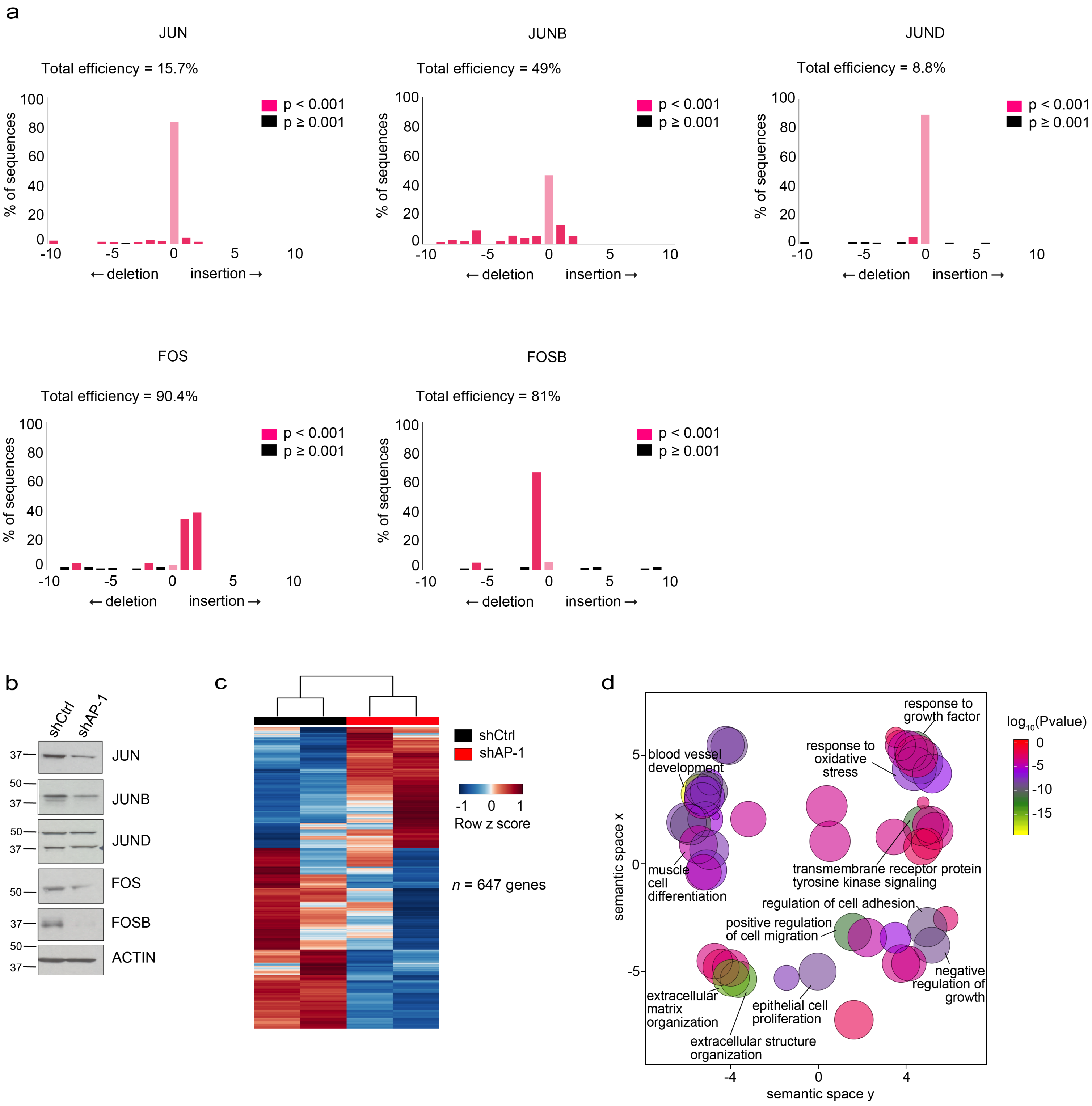

Supplementary Figure 8. AP-1 loss leads to enhancer architecture changes and ECM gene dysregulation. **(a)** CRISPR/Cas9 mediated AP-1 subunit gene editing efficiencies as determined by sequence trace decomposition. **(b)** Western blot of AP-1 subunits in myometrium primary cells transduced with lentiviral hairpins targeting *JUN*, *JUNB*, *JUND*, *FOS* and *FOSB* (shAP-1). Western blot of cells transduced with non-targeting lentiviral hairpin control (shCtrl) are also shown. **(c)** Hierarchically clustered heat map of all differentially expressed genes in AP-1 knockdown primary cells. Gene expression levels relative to the mean expression are shown as row z scores. **(d)** Scatter plot of confidence scores for enriched gene ontologies associated with differentially expressed genes in AP-1 knockdown primary cells, with ontologies clustered by functional similarity in the semantic space.

Patient # in figures	De-identified Patient # in sequencing data	Mutation	Age	Race/ Ethnicity	Hormone treatment
**PT1**	PT848	G44S	49	Black	Progesterone
**PT2**	PT886	G44D	42	White	None
**PT3**	PT916	G44D	51	White	Lo-estrin
**PT4**	PT967	G44D	43	Unknown	None
**PT5**	PT1063	G44D	43	Black	None
**PT6**	PT354	G44D	38	Black	None
**PT7**	PT563	G44S	45	Black	None
**PT8**	PT728	G44D	44	Latino	None
**PT9**	PT758	G44D	48	Black	None
**PT10**	PT845	G44D	48	Black	None
**PT11**	PT1113	G44D	51	Unknown	None
**PT12**	PT1119	G44S	44	White	None
**PT13**	PT1123	G44S	48	Unknown	None
**PT14**	PT1151	G44S	47	Black	None
**PT15**	PTC57	G44D	50	Black	None

Supplementary Table 3. Clinical and biological characteristics of 15 hysterectomy patients from whom tissue samples for this study were obtained.

Oligomer	Sequence (5' to 3')	Experiment
JUN_ F	*5′-GTCCTTCTTCTCTTGCGTGG-3′*	qRT-PCR
JUN_ R	*5′-GGAGACAAGTGGCAGAGTCC-3′*	
JUNB_ F	*5′-AGGCTCGGTTTCAGGAGTTT-3′*	
JUNB_ R	*5′-GAACAGCCCTTCTACCACGA-3′*	
JUND_ F	*5′-CACCTTGGGGTAGAGGAACTG-3′*	
JUND_ R	*5′-CCTCATCATCCAGTCCAACGG-3′*	
FOS _F	*5′-CTACCACTCACCCGCAGACT-3′*	
FOS _R	*5′-GTGGGAATGAAGTTGGCACT-3′*	
FOSB_ F	*5′-CTAGGAGACCCCGAGAGGAG-3′*	
FOSB_R	*5′-ACCAGCACAAACTCCAGACG-3′*	
JUN (TRCN0000039590)	*5′-CGCAAACCTCAGCAACTTCAA-3′*	Short hairpin target sequences
JUNB (TRCN0000232083)	*5′-TCATACACAGCTACGGGATAC-3′*	
JUND (TRCN0000416920)	*5′-GAAGAACAGAGTGTTCGATTC-3′*	
FOS (TRCN0000273940)	*5′-TCTCCAGTGCCAACTTCATTC-3′*	
FOSB (TRCN0000016071)	*5′-GCCGAGTCTCAATATCTGTCT-3′*	
JUN (Hs.Cas9.JUN.1.AA)	*CCATAAGGTCCGCTCTCGGA*	CRISPR/Cas9 crRNA target sequences
JUNB (Hs.Cas9.JUNB.1.AB)	*CGACGACTCATACACAGCTA*	
JUND (Hs.Cas9.JUND.1.AB)	*TGGTGACCAGCCCGTTGGAC*	
FOS (Hs.Cas9.FOS.1.AA)	*GGGCTTCAACGCAGACTACG*	
FOSB (Hs.Cas9.FOSB.1.AA)	*CGTCGACCCCTACGACATGC*	
JUN_ F	*5′-TCCTGGGACTCCATGTCGAT-3′*	gDNA amplification primers
JUN_ R	*5′-TGCGTGCGCTCTTAGAGAAA-3′*	
JUNB_ F	*5′-AAACGACGCCAGGAAAGCTA-3′*	
JUNB_ R	*5′-GGGTGTCACGTGGTTCATCT-3′*	
JUND_ F ***	*5′-CTCAGGTTCGCGTAGACAGG-3′*	
JUND_ R ***	*5′-CTCCATGCAAATGAGCGACG-3′*	
FOS _F	*5′-CAGTTCCCGTCAATCCCTCC-3′*	
FOS _R	*5′-TCTGCGGGTGAGTGGTAGTA-3′*	
FOSB_ F	*5′-TCTCCTTCTCCCTCCCCTTG-3′*	
FOSB_R	*5′-CCTACCCAGGCCAAGTTCTG-3′*	
**MED12 _F (exon 2)**	5′-GCTGGGAATCCTAGTGACCA-3′	
**MED12 _R (exon 2)**	5′-GGCAAACTCAGCCACTTAGG-3′	

Supplementary table 4. DNA sequences used in the current study. *JUND* requires DMSO and betadine for PCR amplification (***).

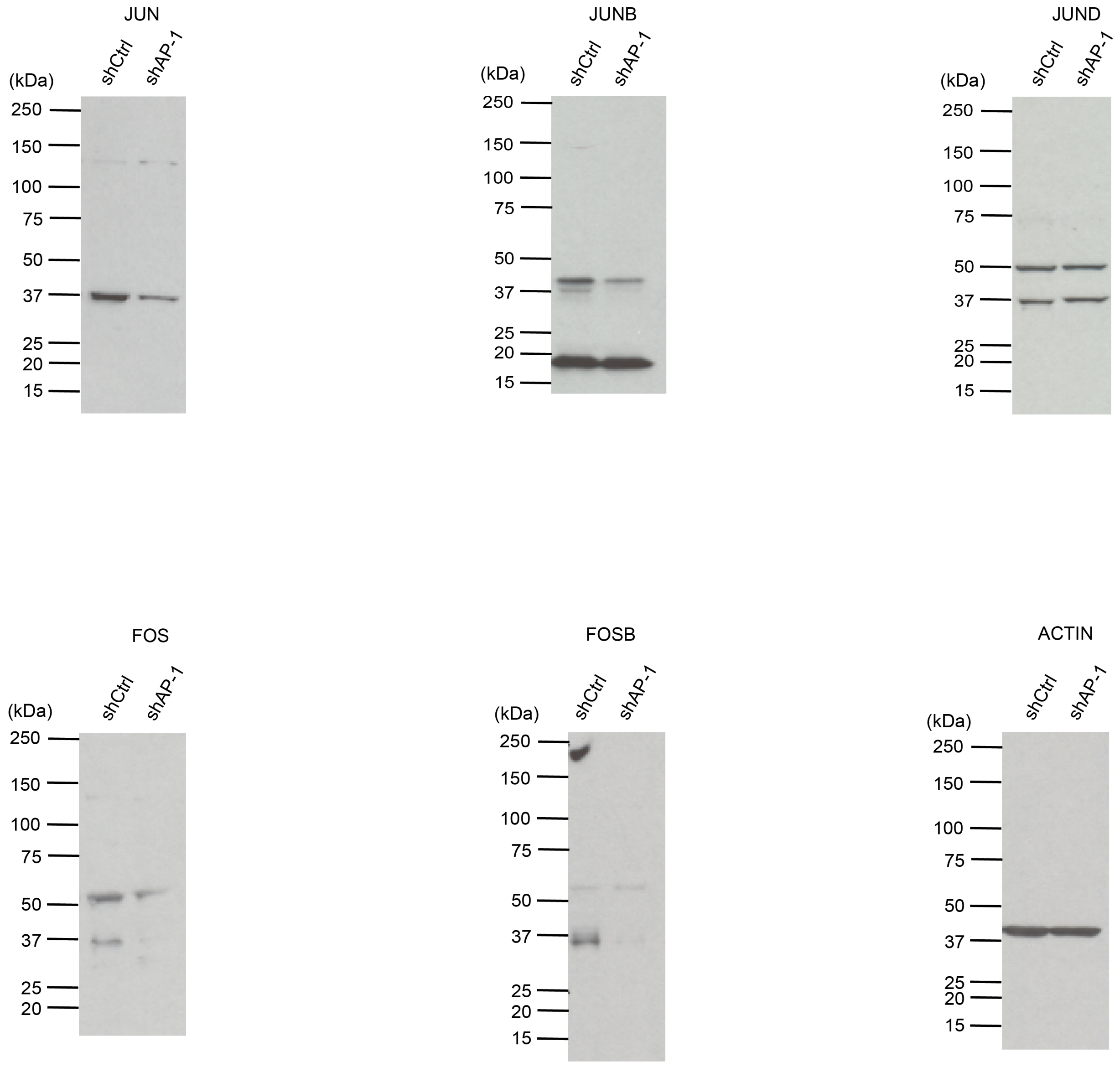

Supplementary Figure 9. Uncropped scan of western blot. Immunoblot of AP-1 subunits in myometrium primary cells transduced with lentiviral hairpins targeting *JUN*, *JUNB*, *JUND*, *FOS* and *FOSB* (shAP-1). Related to supplementary figure 8b.
