# Supplementary material for: Altered chromatin landscape and enhancer engagement underlie transcriptional dysregulation in MED12 mutant uterine leiomyomas
Source: Nat Commun. 2020 Feb 24;11:1019. doi: 10.1038/s41467-020-14701-6 (PMC7040020; doi:10.1038/s41467-020-14701-6)
Supplement: Supplementary file 3 — Description of Additional Supplementary Files [file 41467_2020_14701_MOESM3_ESM.pdf]

Description of Additional Supplementary Files

File Name: Supplementary Data 1

Description: RNA-sequencing alignment statistics.

File Name: Supplementary Data 2

Description: ChIP-sequencing alignment statistics.

File Name: Supplementary Data 3

Description: Promoter capture Hi-C sequencing alignment statistics.

File Name: Supplementary Data 4

Description: Myometrium vs Leiomyoma Tissue differential gene expression enriched gene ontologies.

File Name: Supplementary Data 5

Description: Differential gene expression of enriched gene ontologies for CRISPR/Cas9 mediated AP-1 depletion in HUtSMC.

File Name: Supplementary Data 6

Description: Differential gene expression of enriched gene ontologies for small hairpin mediation AP-1 depletion in primary myometrium cells.
