# Supplementary material for: Altered chromatin landscape and enhancer engagement underlie transcriptional dysregulation in MED12 mutant uterine leiomyomas
Source: Nat Commun. 2020 Feb 24;11:1019. doi: 10.1038/s41467-020-14701-6 (PMC7040020; doi:10.1038/s41467-020-14701-6)
Supplement: Supplementary file 10 — Reporting Summary [file 41467_2020_14701_MOESM10_ESM.pdf]

nature research

Corresponding author(s): Debabrata Chakravarti

Last updated by author(s): Jan 2, 2020

# Reporting Summary

Nature Research wishes to improve the reproducibility of the work that we publish. This form provides structure for consistency and transparency in reporting. For further information on Nature Research policies, see Authors & Referees and the Editorial Policy Checklist.

## Statistics

For all statistical analyses, confirm that the following items are present in the figure legend, table legend, main text, or Methods section.

| n/a | Confirmed | |
|---|---|---|
| ☐ | ☒ | The exact sample size ($n$) for each experimental group/condition, given as a discrete number and unit of measurement |
| ☐ | ☒ | A statement on whether measurements were taken from distinct samples or whether the same sample was measured repeatedly |
| ☐ | ☒ | The statistical test(s) used AND whether they are one- or two-sided<br>*Only common tests should be described solely by name; describe more complex techniques in the Methods section.* |
| ☐ | ☒ | A description of all covariates tested |
| ☐ | ☒ | A description of any assumptions or corrections, such as tests of normality and adjustment for multiple comparisons |
| ☐ | ☒ | A full description of the statistical parameters including central tendency (e.g. means) or other basic estimates (e.g. regression coefficient) AND variation (e.g. standard deviation) or associated estimates of uncertainty (e.g. confidence intervals) |
| ☐ | ☒ | For null hypothesis testing, the test statistic (e.g. $F$, $t$, $r$) with confidence intervals, effect sizes, degrees of freedom and $P$ value noted<br>*Give P values as exact values whenever suitable.* |
| ☒ | ☐ | For Bayesian analysis, information on the choice of priors and Markov chain Monte Carlo settings |
| ☒ | ☐ | For hierarchical and complex designs, identification of the appropriate level for tests and full reporting of outcomes |
| ☒ | ☐ | Estimates of effect sizes (e.g. Cohen's $d$, Pearson's $r$), indicating how they were calculated |

*Our web collection on statistics for biologists contains articles on many of the points above.*

## Software and code

Policy information about availability of computer code

| | |
|---|---|
| Data collection | Not applicable. |
| Data analysis | No custom software or algorithm was used in this study. All software used in this study, including version numbers, are also described in the methods. Software includes: cutadapt, STAR aligner, Bowtie, FastQC, RSeQC, wigToBigWig, featureCounts, Metascape (available online), REVIGO (available online), HOMER, DeepTools, and HiCUP. R packages used in this study include DESeq2, EISA, DiffBind, edgR, CHiCAGO, pheatmaps, ggplot2, LSD and Gviz. |

For manuscripts utilizing custom algorithms or software that are central to the research but not yet described in published literature, software must be made available to editors/reviewers. We strongly encourage code deposition in a community repository (e.g. GitHub). See the Nature Research guidelines for submitting code & software for further information.

## Data

Policy information about availability of data

All manuscripts must include a data availability statement. This statement should provide the following information, where applicable:

- Accession codes, unique identifiers, or web links for publicly available datasets
- A list of figures that have associated raw data
- A description of any restrictions on data availability

All high throughput sequencing data as well as source data underlying figures that support the findings of this study have been made available through the National Center for Biotechnology Information (NCBI) Gene Expression Omnibus (GEO) data repository and can be accessed via accession 'GSE128242 [https://www.ncbi.nlm.nih.gov/geo/query/acc.cgi?acc=GSE128242]'. All data analysis was performed with publicly available software and is listed in the methods.

# Field-specific reporting

Please select the one below that is the best fit for your research. If you are not sure, read the appropriate sections before making your selection.

☒ Life sciences ☐ Behavioural & social sciences ☐ Ecological, evolutionary & environmental sciences

For a reference copy of the document with all sections, see nature.com/documents/nr-reporting-summary-flat.pdf

# Life sciences study design

All studies must disclose on these points even when the disclosure is negative.

| | |
|---|---|
| Sample size | No statistical methods were used to determine sample sizes. |
| Data exclusions | No data were excluded from the analysis. |
| Replication | Biological replicates (i.e. tissue from different patients) were processed in three separate batches and the experimental procedure for each experiment type (RNA-seq/ ChIP-seq/ CHiC) was repeated on separate occasions for each batch to ensure reproducibility of the experimental protocol. However, technical replication for each biological replicate was not attempted. |
| Randomization | Due to the experimental design and goals of this study, all pre-menopause patients undergoing hysterectomies as a treatment for uterine fibroids that were identified to have G44D/S positive tumors were included in this study. |
| Blinding | Due to the experimental design in which tissue samples had to be confirmed to be normal (WT) or MED12 mutant tumors, blinding was not possible during sample preparation or during data collection and analysis. |

# Reporting for specific materials, systems and methods

We require information from authors about some types of materials, experimental systems and methods used in many studies. Here, indicate whether each material, system or method listed is relevant to your study. If you are not sure if a list item applies to your research, read the appropriate section before selecting a response.

## Materials & experimental systems

| n/a | Involved in the study | |
|---|---|---|
| ☐ | ☒ | Antibodies |
| ☐ | ☒ | Eukaryotic cell lines |
| ☒ | ☐ | Palaeontology |
| ☒ | ☐ | Animals and other organisms |
| ☐ | ☒ | Human research participants |
| ☒ | ☐ | Clinical data |

## Methods

| n/a | Involved in the study | |
|---|---|---|
| ☐ | ☒ | ChIP-seq |
| ☒ | ☐ | Flow cytometry |
| ☒ | ☐ | MRI-based neuroimaging |

## Antibodies

| | |
|---|---|
| Antibodies used | All antibodies used in this study and their catalog numbers are also listed in the methods. ChIP-seq antibodies: H3K27Ac (Active motif, cat # 39685, clone 0309, lot #s 21516012 and 14517014), RNAPII (Millipore, cat # 05-952, clone 8WG16, lot # 2839641), FOS (Millipore, cat # 06-341), JUN (abcam, cat # ab31419, lot # GR306615-13), CDK8 and MED12 (Bethyl, cat #s A302-500A, A300-774A respectively).<br>Western blot antibodies: JUN, JUNB (Bethyl, cat #s A302-958A, A302-704A respectively), JUND (Abcam, cat # ab28837, lot # GR121087-3), FOS (ThermoFisher, cat # MA5-15055, lot # UA2699265) and FOSB (Active motif, cat # 40960, lot # 01719012) |
| Validation | - H3K27Ac antibody was validated by the manufacturer and independently validated for specificity: http://www.histoneantibodies.com/FinalArrayData/H3K27ac/<br>- CDK8 antibody was validated by the manufacturer and independently for specificity within our lab group by western blot of CDK8 knockdown cells.<br>- RNAPII and MED12 have been used in previously published ChIP-sequencing studies and were also validated by the manufacturer.<br>- JUN, JUNB, FOS and FOSB antibodies used for western blotting were validated for specificity within our lab group by western blot of knockdown cells. All AP-1 antibodies used in this study were validated by the manufacturer. |

# Eukaryotic cell lines

Policy information about cell lines

| | |
|---|---|
| Cell line source(s) | Human uterine smooth muscle primary cells (HUtSMC). Obtained from American Type Culture Collection (ATCC, cat # PCS-460-011) |
| Authentication | New vial of authenticated primary cells were obtained directly from American Type Culture Collection (ATCC, cat # PCS-460-011). |
| Mycoplasma contamination | New vial of mycoplasma-free primary cells were obtained directly from American Type Culture Collection (ATCC, cat # PCS-460-011). |
| Commonly misidentified lines (See ICLAC register) | No commonly misidentified cell lines were used in this study. |

# Human research participants

Policy information about studies involving human research participants

| | |
|---|---|
| Population characteristics | Patient characteristics are described in the supplementary materials. |
| Recruitment | All pre-menopause patients undergoing hysterectomies as a treatment for uterine fibroids that were identified to have G44D/S positive tumors were included in this study. |
| Ethics oversight | Northwestern University Institutional Review Board. |

Note that full information on the approval of the study protocol must also be provided in the manuscript.

# ChIP-seq

## Data deposition

☒ Confirm that both raw and final processed data have been deposited in a public database such as GEO.

☒ Confirm that you have deposited or provided access to graph files (e.g. BED files) for the called peaks.

| | |
|---|---|
| Data access links *May remain private before publication.* | *For "Initial submission" or "Revised version" documents, provide reviewer access links. For your "Final submission" document, provide a link to the deposited data.* |
| Files in database submission | FASTQ files for MNase ChIP-sequencing of H3K27Ac and high resolution MNase ChIP-sequencing of RNAPII, JUN, FOS, CDK8 and MED12 from 5 myometrium and 5 leiomyoma patient tissue samples. Bigwig files, peak files, and data analysis files are also included. |
| Genome browser session (e.g. UCSC) | *Provide a link to an anonymized genome browser session for "Initial submission" and "Revised version" documents only, to enable peer review. Write "no longer applicable" for "Final submission" documents.* |

## Methodology

| | |
|---|---|
| Replicates | 5 biological replicates |
| Sequencing depth | Sequencing depth of all experiments are provided in the supplementary materials. |
| Antibodies | H3K27Ac (Active motif, cat # 39685, clone 0309, lot # 21516012), RNAPII (Millipore, cat # 05-952, clone 8WG16, lot # 2839641), FOS (Millipore, cat # 06-341), JUN (abcam, cat # ab31419, lot # GR306615-13), CDK8 and MED12 (Bethyl, cat #s A302-500A, A300-774A respectively). |
| Peak calling parameters | Data analysis and peak calling parameters are provided in the methods. |
| Data quality | All assigned peaks were identified based on an FDR < 0.001. As the MNase digestion protocol used in high resolution ChIP enriches for histone or factor-protected DNA, it is inaccurate to consider inputs as background as they are highly biased in favor of protected regions of DNA. As such, standards used to determine data quality in conventional ChIP-sequencing are not applicable in this study. However, all identified peaks have 4-fold higher signal over local background. Detailed peak calling parameters are described in the methods. |
| Software | All ChIP-seq data analysis software and methodology are described in the methods. |
